# Tetramethylpyrazine promotes the proliferation and migration of brain endothelial cells

**DOI:** 10.3892/mmr.2014.2169

**Published:** 2014-04-24

**Authors:** MINGSHUN ZHANG, FENG GAO, FENGMENG TENG, CHUNBING ZHANG

**Affiliations:** 1 Department of Microbiology and Immunology, Nanjing Medical University, Nanjing, Jiangsu 210029, P.R. China; 2 Department of Laboratory Medicine, Jiangsu Province Hospital of TCM, Nanjing, Jiangsu 210029, P.R. China; 3 Basic Medical Sciences, Nanjing University of Chinese Medicine, Nanjing, Jiangsu 210046, P.R. China

**Keywords:** tetramethylpyrazine, endothelial cells, proliferation, soluble FasL, VEGF

## Abstract

The aim of the present study was to investigate the role of tetramethylpyrazine (TMP), one of the alkaloids isolated from the Chinese herb Chuanxiong, on the proliferation and migration of brain endothelial cells. A different dosage of TMP was employed to stimulate the mouse microvascular cell line bEnd.3 *in vitro*. TMP at lower concentrations (0.25 ng/ml), however not at high concentrations (100 ng/ml) could promote the proliferation and migration of endothelial cells, which was further enhanced if combined with soluble Fas ligand (sFasL). TMP alone, or combined with sFasL, increased the autocrine signaling of vascular endothelial growth factor (VEGF) by endothelial cells and TMP improved the expression of Fas on endothelial cells, which may explain the effect of the sFasL. These results provide insight into the underlying mechanisms of the effects of TMP on stroke and other vascular diseases.

## Introduction

Cardiovascular diseases and stroke are among the most important leading causes of morbidity and mortality worldwide. The vascular lumen, either from the peripheral organs or the central nervous system, is covered with endothelial cells. Endothelial cells not only respond to but also produce and release substances that relax or constrict the blood vessels, which may contribute to the development of vascular failure (1). Endothelial cells are also of great importance in disease recovery. The formation of new blood vessels, or angiogenesis, is necessary to fully supply tissues with their metabolic and functional requirements in the long-term. The proliferation and migration of endothelial cells is essential for angiogenesis (2). Systemic administration of human cord blood-derived CD34^+^ cells to immunocompromised mice subjected to stroke 48 h earlier induces neovascularization in the ischemic zone and provides a favorable environment for neuronal regeneration (3). Although CD34^+^ circulating endothelial progenitor cells have the capacity to participate in neovascularization in ischemic tissues (4–5), the outgrowth of pre-existing vasculature is assumed to be indispensable in the postnatal development of neovessels. Thus, the capacity for proliferation and migration in endothelial cells is crucial for the recovery of vascular diseases, including cardiovascular diseases and stroke.

Tetramethylpyrazine (TMP), a biologically active alkaloid isolated from the rhizome of the traditional herbal medicine *Ligusticum walliichi* (Chuanxiong), has been used routinely in China for the treatment of stroke and other vascular diseases. TMP, or its derivatives, was reported to scavenge free radicals, inhibit Ca^2+^ influx (6), increase the transcription of thioredoxin (7) and suppress the inflammatory response (8), thus protecting the neurons in rat ischemic stroke models. The effects of TMP on the proliferation and migration of endothelial cells, however, have not been well explored. Therefore, the present study focused on the proliferation and migration of endothelial cells induced by TMP and its mechanisms with the aim of identifying possible novel targets for the treatment of cardiovascular diseases and stroke.

## Materials and methods

### Endothelial cell proliferation assay

The immortalized mouse brain microvascular endothelial cell line bEnd.3, purchased from the American Type Culture Collection (Manassas, VA, USA), were grown in complete medium consisting of DMEM GlutaMAX, supplemented with 1% penicillin/streptomycin and 10% FCS (Gibco-BRL, Melbourne, Australia).

For the proliferation assay, 1×10^4^ cells in 100 μl complete medium were seeded into each well of a 96-well plate. Following 24 h, different concentrations of TMP (Chinese National Institute for the Control of Pharmaceutical and Biological Products, Beijing, China) or combined with soluble FasL (Sigma, St. Louis, MO, USA) were used to stimulate endothelial cells for 48 h. At the end of the cell culture, 20 μl of CCK-8 solution (5 mg/ml; Dojindo, Kumamoto, Japan) was added into each well and cells were incubated for an additional 4 h. Cell proliferation was measured with a microplate reader at 450 nm and the proliferation index was calculated as follows: (OD450 in the presence of TMP - OD450 in the blank control)/OD450 in the blank control × 100%.

### Endothelial cell migration assay

The migration of endothelial cells were assayed in an *in vitro* scraping experiment as described elsewhere. Briefly, cells were grown in a 6-well plate. When the cell confluence reached 60%, cells were injured by a deliberate scratch with a 1,000 μl pipette tip and stimulated with TMP alone (0.25 ng/ml) or combined with sFasL (0.16 ng/ml). Cells were washed with PBS softly and images were captured, 24 h after injury. Distance of the scratch was calibrated in triplicate in a blinded manner.

### ELISA for VEGF

Cells grown in the 6-well plate were treated with TMP alone or combined with soluble FasL for 72 h. The supernatant was harvested and stored in −80°C until further analysis. ELISA for the VEGF was performed according to the manufacturer’s instructions (R&D Systems, Minneapolis, MN, USA). Briefly, 50 μl assay diluents were added to each well, followed by the addition of 50 μl standard, control or cell culture supernatant and the plate was incubated at room temperature for 2 h. Following incubation, each well was aspirated and washed five times and 100 μl conjugated secondary antibody was added into each well for 2 h at room temperature. Following extensive washing, 100 μl of substrate solution was added to each well for 30 min at room temperature. Finally, 100 μl of stop solution was added to each well and the absorbance was read at 450 nm on an ELISA reader (Bio-Rad, Hercules, CA, USA) within 30 min. All readings were repeated at least three times.

### Western blotting for the Fas protein

Cells grown in the 6-well plate were treated with TMP alone or combined with soluble FasL for 72 h. Following being washed twice with ice-cold PBS, an ice-cold cell lysis buffer was added to the cells and the solution was passed through a pipette several times. The homogenates were centrifuged at 4°C and 18,188 × g for 30 min. Proteins were measured using the BCA assay. Equal amounts of protein samples were separated using electrophoresis on 10% sodium dodecyl sulfate-polyacrylamide gels (SDS-PAGE) and transferred onto polyvinylidene difluoride (PVDF) membranes. The membranes were inhibited with Tris-buffered saline Tween-20 (TBST) containing 5% (w/v) skimmed milk at room temperature for 2 h. The membranes were incubated with anti-Fas (dilution 1:2,000; KeyGen Biotech, Nanjing, Jiangsu, China) overnight at 4°C. Following washing, the membranes were incubated with HRP-conjugated secondary antibodies for 1 h at room temperature. Antigen was detected using enhanced chemiluminescence (Pierce Biotechnology, Inc., Rockford, IL, USA). All the samples were normalized to β-actin.

### Imaging and statistical analysis

Blots were scanned and quantified using Image J software. Blots were quantified as follows: the value attained for each sample was divided by the value of the corresponding β-actin and then expressed as a normalized ratio.

Data are expressed as the mean ± SEM. Intergroup comparisons were performed using one-way analysis of variance (ANOVA) for multiple comparisons followed by Fisher’s protected least significant difference (PLSD). P<0.05 was considered to indicate a statistically significant difference.

## Results

### Proliferation of endothelial cells stimulated by low-dosage TMP

**Figure 1:**
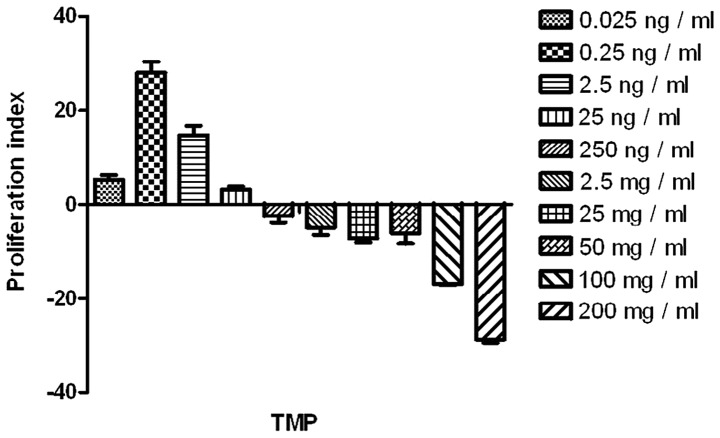
TMP stimulated the proliferation of endothelial cells. Different doses of TMP, ranging between 0 ng/ml (blank control), 0.025 ng/ml and 200 ug/ml, were tested for its effect on the proliferation of endothelial cells. Notably, only TMP at the lower dose (0.025 ng/ml and 0.25 ng/ml) could stimulate proliferation, while, overdose (100 ug/ml and 200 ug/ml) significantly inhibited cell growth. ^*^P<0.05, compared with the blank control. TMP, tetramethylpyrazine.

TMP at a low-dosage, ranging from 0.025 ng/ml to 0.25 ng/ml, promoted the proliferation of the endothelial cells significantly (compared with the blank control; P<0.05). TMP at a higher dosage, particularly >100 ng/ml, impaired the endothelial cells (Fig. 1). Thus, TMP at 0.25 ng/ml was selected for the analysis of the combined effects with sFasL.

### Effect of sFasL on the TMP-stimulated proliferation of endothelial cells

**Figure 2:**
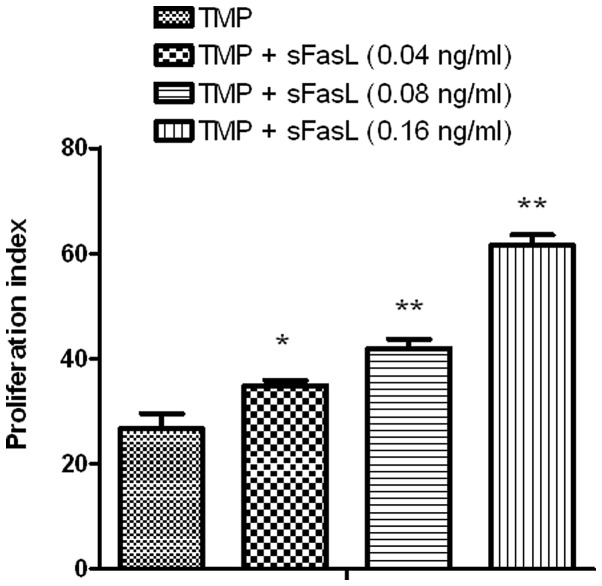
sFasL aided TMP stimulation of the proliferation of endothelial cells. TMP at 0.25 ng/ml alone or combined with sFasL, was used for the cell culture. sFasL significantly enhanced the TMP-induced proliferation of the endothelial cells, which was dose dependent. ^*^P<0.05, compared with the blank control; ^**^P<0.05, compared with the TMP alone group. sFasL, soluble Fas ligand; TMP, tetramethylpyrazine.

As depicted in Fig. 1, TMP at 0.25 ng/ml could stimulate the division of endothelial cells and sFasL (0.16 ng/ml) could also stimulate the proliferation of endothelial cells via the Fas-FasL pathway. Notably, compared with TMP alone, TMP combined with sFasL was a more powerful stimulant for the proliferation of endothelial cells (P<0.05; Fig. 2).

### TMP combined with sFasL enhances the migration of endothelial cells

**Figure 3:**
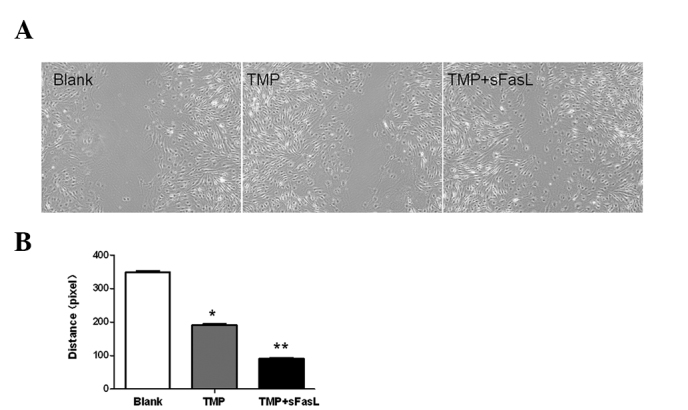
TMP combined with sFasL enhanced the migration of endothelial cells. (A) Either TMP alone or combined with sFasL increased the migration of endothelial cells. Representative images for each group, repeated at least four times. (B) Quantification of the migration of endothelial cells; ^*^P<0.05, compared with the blank control; ^**^P<0.05, compared with the TMP alone group. sFasL, soluble Fas ligand; TMP, tetramethylpyrazine.

TMP promoted the proliferation of endothelial cells. Endothelial cell migration is also essential for angiogenesis. In the present study, TMP could also enhance the migration of endothelial cells, which is more evident if combined with sFasL (P<0.05; Fig. 3).

### Fas expression increases upon stimulation of TMP

**Figure 4:**
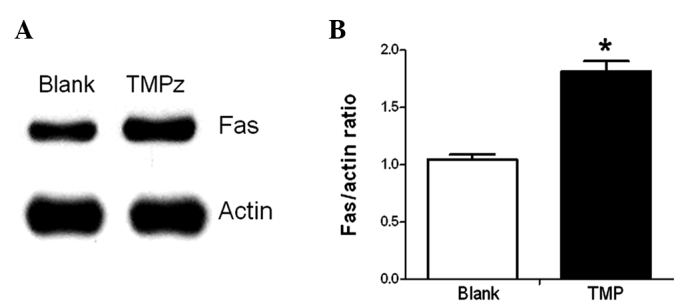
Fas expression increased upon the stimulation of TMP. bEnd.3 endothelial cells were stimulated with TMP (0.25 ng/ml) and the cell lysate was harvested for the quantification of Fas. (A) Western blotting for the blank control or the TMP group; β-actin was used as an internal control. (B) Quantification of Fas was normalized to β-actin and is expressed as the mean ± SEM (n=4); ^*^P<0.05, compared with the blank control. TMP, tetramethylpyrazine.

TMP and sFasL could promote the proliferation and migration of endothelial cells. In addition, TMP also increased the expression of Fas on endothelial cells (P<0.05; Fig. 4).

### TMP promotes the autocrine signaling of VEGF by endothelial cells

**Figure 5:**
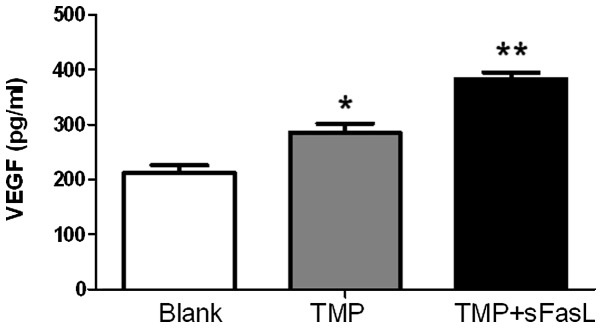
VEGF secretion by endothelial cells. TMP increased the autocrine signaling of VEGF by endothelial cells, which was further improved by the combination with sFasL. ^*^P<0.05, compared with the blank control; ^**^P<0.05, compared with the TMP alone group. TMP, tetramethylpyrazine; VEGF, vascular endothelial growth factor; sFasL, soluble Fas ligand.

The proliferation and migration of endothelial cells were mediated by VEGF. Accordingly, the endothelial cells secreted significantly more VEGF upon the stimulation of TMP. sFasL combination further increased the autocrine signaling of VEGF (Fig. 5).

## Discussion

Previously, it was verified that TMP suppressed the production of nitric oxide in human umbilical vein endothelial cells (HUVECs) stimulated with TNF-α (9). TMP also decreased the expression of intracellular adhesion molecule-1 and heat shock protein 60, suggesting its anti-inflammatory role in endothelial cells (9). As a reactive oxygen species antagonist, TMP protected the rat pulmonary microvascular endothelial cells from hypoxia and TMP-treated animals demonstrated less pulmonary vascular leakage compared with those exposed to hypoxia alone (10). It was also reported that TMP, or its derivatives, suppressed the apoptosis of HUVECs induced by hydrogen peroxide (11). Whether TMP could modulate the proliferation and migration of endothelial cells, however, is largely unknown.

The dosage effects of TMP on endothelial cells were first investigated. TMP at a rather lower dosage (0.025 ng/ml or 0.25 ng/ml) significantly promoted the proliferation of the endothelial cells, while TMP at a concentration >100 ng/ml appeared negative for the proliferation of endothelial cells in the physiological condition. Plasma concentration may affect the efficiency of TMP in the treatment of stroke (12). The results presented in the present study suggest that TMP overdose may also be harmful, which is occasionally reported in clinical practice.

TMP promoted not only the proliferation but also the migration of the endothelial cells, which was further improved by sFasL. Considering the Fas-FasL pathway in angiogenesis, it may be valuable to detect whether TMP could regulate the Fas-FasL pathway in endothelial cells. Fas-FasL ligation induced proliferation by recruiting the Fas-associated death domain protein and the Flice-like inhibitory protein (FLIP). FLIP further recruited and activated the downstream molecules TNF-receptor-associated factor and nuclear factor κB (NF-κB), which eventually promoted the proliferation of the cells. In the photoreceptor cells damaged by N-methyl-N-nitrosourea, TMP increased the expression of NF-κB and suppressed the apoptosis of cells (13). NF-κB is one of the downstream molecules of the Fas-FasL pathway. As verified in the present study, TMP also directly upregulated the expression of Fas, which may expand our understanding of the underlying mechanisms of TMP on endothelial cells.

In summary, TMP promoted the proliferation and migration of endothelial cells, which was further improved by sFasL. The positive roles of TMP on the endothelial cells was partially dependent on the increased expression of Fas and enhanced secretion of VEGF. Further study may elucidate whether Fas could be a novel target in the treatment of vascular diseases and stroke.

## References

[1] Hirase T, Node K (2012). Endothelial dysfunction as a cellular mechanism for vascular failure. Am J Physiol Heart Circ Physiol.

[2] Fahmy RG, Dass CR, Sun LQ (2003). Transcription factor Egr-1 supports FGF-dependent angiogenesis during neovascularization and tumor growth. Nat Med.

[3] Taguchi A, Soma T, Tanaka H (2004). Administration of CD34^+^cells after stroke enhances neurogenesis via angiogenesis in a mouse model. J Clin Invest.

[4] Asahara T, Masuda H, Takahashi T (1999). Bone marrow origin of endothelial progenitor cells responsible for postnatal vasculogenesis in physiological and pathological neovascularization. Circ Res.

[5] Asahara T, Murohara T, Sullivan A (1997). Isolation of putative progenitor endothelial cells for angiogenesis. Science.

[6] Sun Y, Yu P, Zhang G (2012). Therapeutic effects of tetramethylpyrazine nitrone in rat ischemic stroke models. J Neurosci Res.

[7] Zhu XL, Xiong LZ, Wang Q (2009). Therapeutic time window and mechanism of tetramethylpyrazine on transient focal cerebral ischemia/reperfusion injury in rats. Neurosci Lett.

[8] Liao SL, Kao TK, Chen WY (2004). Tetramethylpyrazine reduces ischemic brain injury in rats. Neurosci Lett.

[9] Wu HJ, Hao J, Wang SQ (2012). Protective effects of ligustrazine on TNF-alpha-induced endothelial dysfunction. Eur J Pharmacol.

[10] Zhang L, Deng M, Zhou S (2011). Tetramethylpyrazine inhibits hypoxia-induced pulmonary vascular leakage in rats via the ROS-HIF-VEGF pathway. Pharmacology.

[11] Zhai L, Zhang P, Sun RY (2011). Cytoprotective effects of CSTMP, a novel stilbene derivative, against H2O2-induced oxidative stress in human endothelial cells. Pharmacol Rep.

[12] Ho WK, Wen HL, Lee CM (1989). Tetramethylpyrazine for treatment of experimentally induced stroke in Mongolian gerbils. Stroke.

[13] Yang JN, Chen JM, Luo L (2005). Tetramethylpyrazine protected photoreceptor cells of rats by modulating nuclear translocation of NF-kappaB. Acta Pharmacol Sin.

